# Mineralocorticoid receptor antagonists in cardiovascular translational biology

**DOI:** 10.1097/XCE.0000000000000289

**Published:** 2023-08-21

**Authors:** Robert J. Chilton, José Silva-Cardoso

**Affiliations:** a Department of Medicine, Janey & Dolph Briscoe Division of Cardiology, Long School of Medicine, UT Health San Antonio, San Antonio, Texas, USA; b Heart Failure and Transplant Clinic, Cardiology Service, São João University Hospital Centre, Porto, Portugal

**Keywords:** cardiovascular outcomes, mineralocorticoid receptor antagonists, translational biology

## Abstract

This review examines the role of mineralocorticoid receptor antagonists (MRAs) in cardiovascular biology and the molecular mechanisms involved in mineralocorticoid receptor antagonism. The data discussed suggest that MRAs can play an important role in decreasing the impact of inflammation and fibrosis on cardiorenal outcomes. Evidence derived from major randomized clinical trials demonstrates that steroidal MRAs reduce mortality in patients with heart failure and reduced ejection fraction. Initial positive findings observed in patients with chronic kidney disease and type 2 diabetes (T2D) indicate the possible mechanisms of action of nonsteroidal MRAs, and the clinical benefits for patients with cardiorenal disease and T2D. This article supports the application of basic science concepts to expand our understanding of the molecular mechanisms of action involved in pathophysiology. This approach encourages the development of treatment options before diseases clinically manifest.

Video Abstract: http://links.lww.com/CAEN/A42

## Introduction

Fibrosis is a major component of ventricular remodeling observed in patients with heart failure [1], and targeting fibrosis is an important therapeutic approach in heart failure management. However, there are limited treatment options for addressing fibrosis and inflammation associated with cardiovascular diseases (CVDs). Over the past two decades, research in this area has targeted specific disease phenotypes, such as hypertension, heart–kidney–liver failure and aging-related vascular stiffness. Endothelial dysfunction [2] and inflammation [3] are key underlying pathologies in CVDs, where inappropriate mineralocorticoid receptor activation plays a significant role. Together with oxidative stress and impaired cardiac calcium signaling, these consequences of mineralocorticoid receptor activation have been shown in preclinical studies to be attenuated with mineralocorticoid receptor antagonists (MRAs).

Although the mechanisms of action of MRAs are not fully understood, first-generation MRAs such as spironolactone [4,5] and eplerenone [6] demonstrated positive clinical benefits in patients with heart failure in the RALES [5], EPHESUS [7] and EMPHASIS-HF [8] trials, including reductions in hospitalizations for heart failure [5,7,8] and in all-cause mortality. Results from the more recent FIDELIO-DKD [9] and FIGARO-DKD [10] trials investigating the nonsteroidal MRA finerenone, have shown consistent cardiovascular improvements in patients with type 2 diabetes (T2D) and chronic kidney disease (CKD).

The aim of this review is to discuss the role of MRAs in CVDs, considering their interaction with the mineralocorticoid receptor when it becomes overactivated. As such, this article will explore how the binding abilities of nonsteroidal MRAs, such as finerenone [9,11–14] and esaxerenone [15,16], impact selectivity and ability to bind to the mineralocorticoid receptor compared with those of steroidal MRAs. Evidence supporting a lower risk of hyperkalemia with nonsteroidal MRAs compared with steroidal MRAs will also be discussed [9,11,12].

Eplerenone and spironolactone are currently approved by the US Food and Drug Administration (FDA) as fourth-line treatments for heart failure and will be examined alongside finerenone, the only nonsteroidal MRA currently approved by the FDA [17] and the European Medicines Agency (EMA) [18]. Other novel nonsteroidal MRAs [19–21] in earlier stages of clinical development will also be evaluated, noting key differences between the two classes of MRAs.

## The development of mineralocorticoid receptor antagonists for the treatment of cardiovascular disease

### Molecular basis of mineralocorticoid receptor being a driver of cardiac remodeling

Our understanding of the role of the mineralocorticoid receptor and its main ligand, aldosterone, in CVD has evolved over the past 30 years. As research has advanced, so has our knowledge of the role of aldosterone. It is one of the physiologic ligands for mineralocorticoid receptor and raises blood pressure primarily by affecting the kidney vasculature and central nervous system. Aldosterone is the main mineralocorticoid hormone. It serves a fundamental role in the control of extracellular volume homeostasis by stimulating renal sodium reabsorption and potassium excretion [22].

**Fig. 1:**
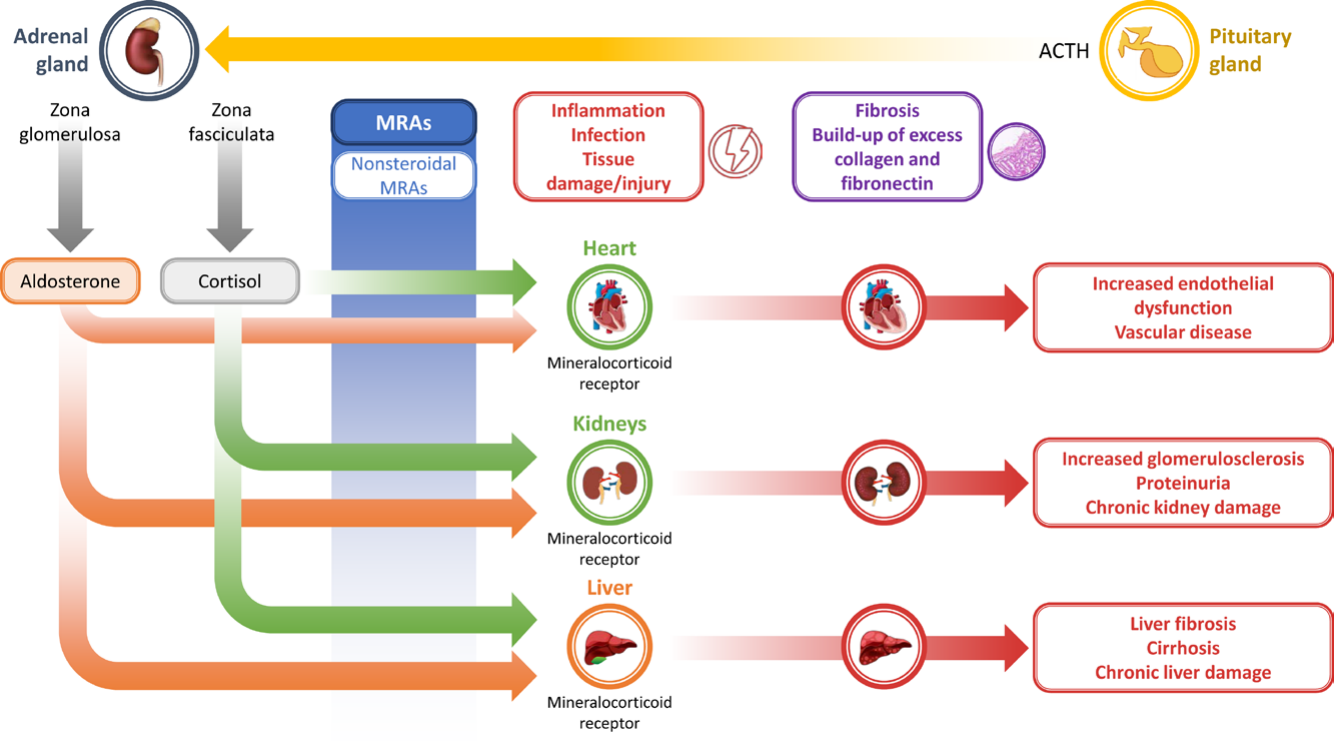
**Mechanisms of action of finerenone.** Overactivation of the mineralocorticoid receptor may lead to inflammation and fibrosis of several organs, causing vascular dysfunction to the heart, kidneys, and liver. It is important to note that inappropriate mineralocorticoid receptor activation by aldosterone and cortisol does not induce equivalent pathology in all three tissue types. Finerenone competes for aldosterone binding sites and decreases blood pressure and aldosterone-mediated gene expression. It has been shown to reduce interstitial fibrosis and tissue damage. ACTH, adrenocorticotropic hormone; MRA, mineralocorticoid receptor antagonist.

Aldosterone acts on the mineralocorticoid receptor in the epithelial cells of the kidney [22]. However, the mineralocorticoid receptor is also expressed in numerous cell types and tissues, including the heart and vascular tissues [23], and is a driver of cardiac remodeling [22]. Vascular tissues express 11-beta-hydroxysteroid dehydrogenase 2, whereas the heart does not [24,25]. Glucocorticoids and aldosterone can activate the mineralocorticoid receptor in the heart (Fig. 1) [23,26]. Mineralocorticoid receptor activation impacts cardiovascular regulation as well as metabolic function through mechanisms such as oxidative stress, inflammation, interstitial fibrosis and endothelial dysfunction [26].

Fibrosis is a critical aspect of tissue repair that arises after tissue injury [27] and can be viewed as a pathologic aspect of most chronic inflammatory diseases [28]. Fibrosis is defined by the buildup of excess extracellular matrix components, such as collagen and fibronectin [28]. In nonpathological tissue repair, accumulation of connective tissue through fibrosis maintains tissue architecture [27,28]. Progressive fibrosis, however, indicates disease and leads to scarring, function impairment and organ damage [27], and can affect all tissues [28] including the myocardium, arteries, kidney and liver (Fig. 1). There is increasing evidence that overactivation of the mineralocorticoid receptor encourages inflammation and fibrosis, impacting the progression of CKD and CVD [29] (Fig. 1), which can affect patient morbidity and mortality associated with these diseases [30].

**Fig. 2:**
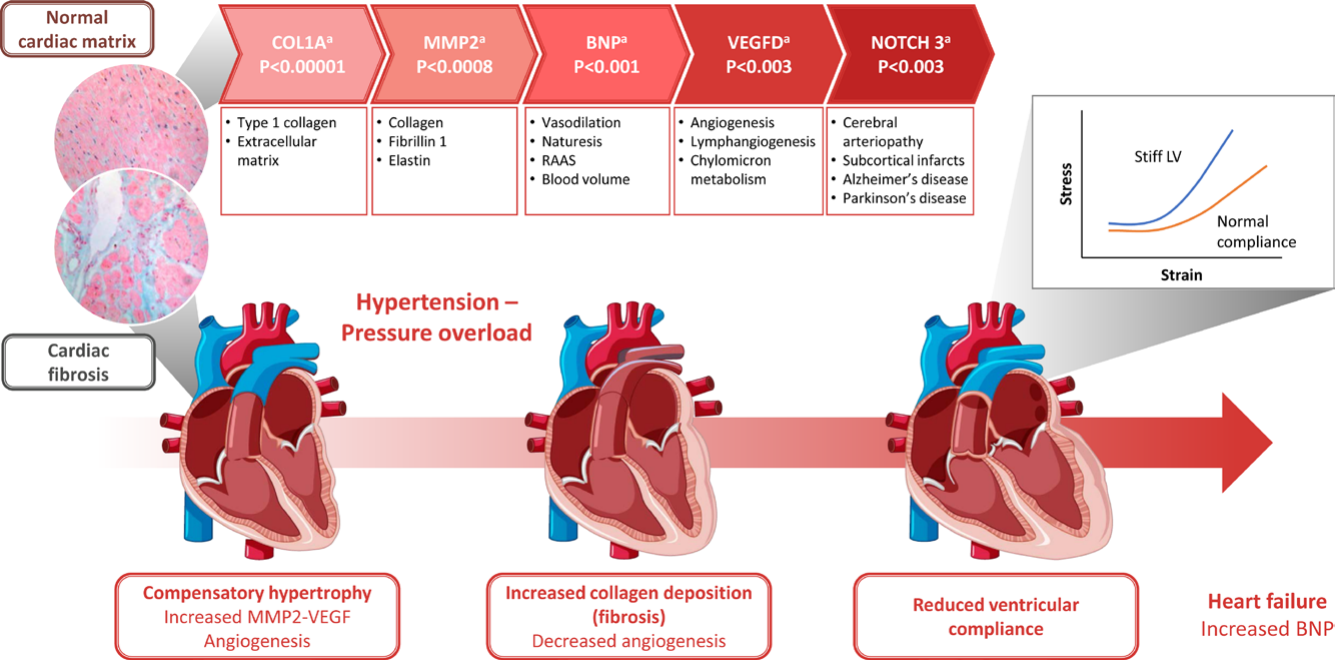
**Five major plasma proteins that are decreased with spironolactone in humans.** This figure is a translational view of basic to clinical medicine. The systolic function is preserved in the stiff, poorly compliant LV at the expense of diastolic function. Diastolic dysfunction becomes increasingly common with diabetes, and heart failure with preserved ejection fraction. The electron micrograph images show normal heart collagen and the development of thick cardiac fibrosis in poorly compliant LV. Figure electron micrograph images included from Chute *et al*. J Cardiovasc Dev Dis 2019;6(4):35 [31]. This is an open-access article distributed under the Creative Commons Attribution License, which permits unrestricted use, distribution and reproduction in any medium, provided the original work is properly cited. ^a^Changes in plasma protein-coding genes with spironolactone treatment from Ferreira JP, *et al*. JACC Heart Fail 2021;9(4):268-277 [32]. BNP, brain natriuretic peptide; LV, left ventricle; MMP2, matrix metalloproteinase 2; RAAS, renin–angiotensin–aldosterone system; VEGF, vascular endothelial growth factor.

The culmination of mineralocorticoid receptor activation in various cell types [vascular smooth muscle cells (VSMCs), endothelial cells, macrophages, cardiomyocytes] contributes to the development of cardiac diastolic dysfunction and heart failure [23]. In endothelial cells, mineralocorticoid receptor contributes to elevated levels of reactive oxygen species and increased oxidative stress, which is associated with vascular inflammation [26] as well as coronary microvascular dysfunction [23]. In VSMCs, mineralocorticoid receptor activation results in increases in smooth muscle cell contraction and oxidative stress [23]. Mineralocorticoid receptor-mediated actions in macrophages increase macrophage activation, chemotaxis and vascular infiltration [23]. Vascular dysfunction in the form of vascular fibrosis can result in reduced elasticity and stiffening (Fig. 1) [26]. Vascular stiffness due to increased accumulation of interstitial collagen is clinically related to fibrosis in many vascular diseases, such as hypertension [23]. In hypertensive heart disease, increased accumulation of interstitial collagen leads to increased left ventricular stiffness, chamber remodeling and ventricular, atrial diastolic and systolic dysfunction (Figs. 1 and 2) [33,34]. Other organs may also experience significant functional impairment owing to reduced blood vessel compliance, seen particularly in the elderly as aging progresses [35,36]. Changes in collagen synthesis and degradation result in the buildup of collagen in blood vessel walls, deterioration in vascular compliance and endothelial dysfunction, which in turn increase the cardiovascular risk in individuals with hypertension (Figs. 1 and 2) [35,36]. Mineralocorticoid receptor activation in cardiomyocytes [23] contributes to overactive fibrosis, which may also lead to unfavorable cardiac remodeling in patients following myocardial infarction, myocardial dysfunction and the development of heart failure [37].

### The molecular structure of mineralocorticoid receptor has impacted the evolution of mineralocorticoid receptor antagonists

Aldosterone was first identified as a mineralocorticoid in 1954 [38], and the role of excessive aldosterone secretion in heart failure and liver cirrhosis with volume overload was soon realized [39]. This led to the discovery by Kagawa *et al*. [40] in 1957 of the first MRA molecule, spironolactone. An oral, bioavailable form of spironolactone known as SC-9420 [41] was approved by the FDA in 1960 for primary hyperaldosteronism and edema of heart failure or cirrhosis. Spironolactone remains approved for the management and treatment of hypertension and heart failure.

The initial development of spironolactones reportedly stemmed from an effort to combine structures of progesterone (an endogenous aldosterone antagonist) and digitoxin [6,42]. As a result, spironolactones were soon recognized as progesterone agonists with anti-androgenic properties [43]. However, the clinical implications of the nonspecific action of spironolactones were first reported in the form of painful gynecomastia [44]. Endocrine dysfunction associated with spironolactone included impotence in men and menstrual irregularities and sore breasts in women [45]. These observations were followed by extensive research aimed toward the development of spironolactones devoid of such side effects. This process led to the development of mexrenone [46], spirorenone [47] and mespirenone [48].

Differences exist between nonsteroidal and steroidal MRAs, not only in their structure but also in their mode of mineralocorticoid receptor antagonism. Preclinical data have shown that the nonsteroidal MRA finerenone is able to exert key antifibrotic effects through its selectivity and nuclear recruitment. It has been shown to demonstrate greater selectivity for the mineralocorticoid receptor than eplerenone and spironolactone while being equally as potent [6]. The risk of hyperkalemia is a key clinical consideration in MRA treatment. The molecular structure of nonsteroidal MRAs in terms of their tissue distribution could provide evidence for a potential link to reduced risk of hyperkalemia [6,13,14].

### Preclinical studies evaluating nonsteroidal mineralocorticoid receptor antagonists in cardiovascular fibrosis reduction

Evidence indicates that mineralocorticoid receptor is implicated in cardiovascular pathophysiology and this has led to studies of the effects of mineralocorticoid receptor blockade. Susic *et al*. [49] found that long-term mineralocorticoid receptor blockade using eplerenone in male, 22-week-old, spontaneously hypertensive rats improved left ventricular diastolic function and coronary hemodynamics. This improvement was associated with an enhancement in cardiac function, independent of changes in blood pressure [49]. In recent decades, advances in research, such as cloning of the human mineralocorticoid receptor and sophisticated robotics (ultra-high throughput screening), have accelerated the development of nonsteroidal MRAs [6,50]. As a result, dihydropyridine calcium channel blockers were determined to have mineralocorticoid receptor antagonistic effects [51], and this observation led to the development of dihydronaphthyridine finerenone (previously known as BAY 94-8862).

Preclinical studies investigating antifibrotic activity revealed that finerenone exhibited higher potency/efficacy and inverse agonism when compared with eplerenone in mineralocorticoid receptor transcriptional co-factor binding assays [52]. Differential mineralocorticoid receptor co-factor modulation has been reported and suggests that finerenone may have properties that contribute to the lessening of cardiac fibrosis effects observed in mice models [52]. Finerenone was also shown to improve cardiovascular dysfunction in ovariectomy-induced left ventricular diastolic dysfunction with preserved ejection fraction [53].

Other nonsteroidal MRAs being investigated in preclinical animal models include the nonsteroidal MRA KBP-5074 (KBP Biosciences; Princeton, New Jersey, USA), which was shown to restrict albuminuria in aldosterone-induced injury in rat models [54]. The main aim of the study mentioned was to compare KBP-5074 with the classical steroidal MRA eplerenone. However, further investigations comparing other nonsteroidal MRAs, such as esaxerenone and finerenone, would offer further insight into the efficacy and safety of this compound [14,54]. Furthermore, these favorable mechanisms of action and characteristics support clinical applications of newer nonsteroidal MRAs in cardiovascular biology.

## The impact of mineralocorticoid receptor antagonist utilization in cardiovascular diseases

Once it was discovered that myocardial fibrosis could be prevented through mineralocorticoid receptor blockade [55,56], the medical community began using spironolactone therapy to improve long-term prognosis in patients with heart failure with reduced ejection fraction (HFrEF), rather than just as a potassium-sparing diuretic. Spironolactone was also proposed to be the ‘renal aspirin’, considering its beneficial effects in terms of preventing proteinuria [4]. Forty years later, the RALES trial demonstrated the efficacy of spironolactone in improving ‘hard’ clinical outcomes and long-term prognosis in patients with HFrEF, including a significant 3% absolute reduction in the risk of sudden cardiac death (*P* = 0.02) [5].

Eplerenone, initially known as the epoxy derivative of mexrenone, is a well-known second-generation MRA [6]. Despite its short half-life and low affinity to the mineralocorticoid receptor, eplerenone was shown to be associated with a significant reduction in cardiovascular deaths in the EPHESUS trial, which involved patients with acute coronary syndrome with left ventricular dysfunction [7]. In the EMPHASIS trial, eplerenone reduced the risk of death from cardiovascular causes or hospitalization for heart failure among patients with HFrEF and mild symptoms compared with placebo [8].

In clinical studies involving MRAs, reductions have been observed in both *N*-terminal pro-B-type natriuretic peptide and procollagen type 1 C-terminal propeptide, without changes in procollagen type 3 *N*-terminal peptide [57]. These findings suggest that MRAs may have a favorable impact on collagen turnover (reducing synthesis and increasing degradation) with improved cardiac remodeling [57] (Fig. 1). The greatest impact observed in the HOMAGE trial, which investigated the effects of spironolactone on fibrosis and cardiac function in people with increased risk of developing heart failure was the change in vascular wall fibrosis through a reduction in collagen type 1 alpha 1 chain (COL1A1) [57]. In addition, it has been demonstrated that MRAs reduce endothelin 1 production and oxidative stress [58]. Proteomic analyses assessing the effect of spironolactone on plasma protein biomarkers indicated that spironolactone reduced levels of collagen metabolism biomarkers (COL1A1, matrix metalloproteinase 2 and brain natriuretic peptide) [32] (Fig. 2). Following 1 month of spironolactone treatment, levels of two other plasma protein biomarkers associated with increased risk of CVD – vascular endothelial growth factor D (VEGFD) and neurogenic locus notch homolog protein 3 (NOTCH3) [32] – also decreased. VEGFD is a powerful lymphangiogenic and angiogenic growth factor [59], and important for endothelial cell growth, while NOTCH3 has been found to affect cell proliferation and apoptosis [32]. Though initial data are promising, the potential impact of fibrosis suppression in CVDs such as heart failure, and in other solid organs, will require more extensive research.

### Clinical evidence for nonsteroidal mineralocorticoid receptor antagonists in cardiovascular biology

The development of nonsteroidal MRAs has been an important advance in cardiovascular translational biology. The list of emerging compounds in this category includes KBP-5074 (KBP Biosciences; phase 2), esaxerenone (Daiichi Sankyo, Tokyo, Japan; phase 3) [60] and finerenone (Bayer AG, Leverkusen, Germany; phase 3) [9,11,61].

Esaxerenone is approved for the treatment of hypertension in Japan [19]. Various other unapproved nonsteroidal MRAs are currently under investigation, including KBP-5074, which is being studied as a treatment for lowering blood pressure in patients with CKD [20,21], and apararenone, which is being investigated in patients with kidney disease and diabetes [62]. Clinical differences between nonsteroidal MRAs, such as association with hypotension and hyperkalemia, may appear to exist, but direct comparisons between compounds are required for these to be truly determined.

The nonsteroidal MRA finerenone is approved by the FDA [17] to reduce the risk of cardiovascular death, non-fatal myocardial infarction and hospitalization for heart failure in adult patients with CKD and T2D. This nonsteroidal MRA is also approved and indicated for the treatment of CKD associated with T2D in adults by the EMA [18]. Finerenone differs from the steroidal MRAs spironolactone (Fig. 2) and eplerenone in its mechanism of action of mineralocorticoid receptor antagonism, pharmacokinetics and effect on inflammation and fibrosis in models of cardiac fibrosis and CKD (Fig. 1 and 2) [30]. Finerenone has a short half-life and no active metabolites, whereas spironolactone is a prodrug with multiple active metabolites with long half-lives; eplerenone has no active metabolites but has a half-life of 4–6 h [30].

The FIDELIO-DKD and FIGARO-DKD trials investigated the efficacy and safety of finerenone in reducing kidney failure and cardiovascular outcomes in patients with CKD and T2D [9,10]. In FIDELIO-DKD, finerenone treatment resulted in lower risk of CKD progression and cardiovascular events compared with placebo [9]. Given that the majority of patients in FIDELIO-DKD were receiving an angiotensin-converting enzyme (ACE) inhibitor or angiotensin receptor blocker (ARB), this was the first study to show the validity of using the combination of two renin–angiotensin–aldosterone system (RAAS) inhibitors in reducing hard kidney and cardiovascular outcomes [9,63]. By contrast, in the ONTARGET, VA-NEPHRON-D, and ALTITUDE trials, which involved RAAS inhibition in combination with non-MRA-based treatments, increased risk of serious adverse events was observed with no significant clinical benefit [63].

The FIGARO-DKD trial built upon the findings from FIDELIO-DKD, and together the two trials covered patient populations with multiple comorbidities. These patients had varying degrees of CKD in T2D [9,10], meaning that they were at high risk for both renal and cardiovascular events. The prognosis benefit of finerenone was observed early in FIDELIO-DKD [9,61], and was maintained for the duration of the trial; kidney outcome benefits were evident at 12 months, and cardiovascular outcome benefits were observed as early as at 1 month [9,30]. FIGARO-DKD demonstrated consistent cardiovascular benefits of finerenone independent of baseline urinary albumin : creatinine ratio and estimated glomerular filtration rate (eGFR) [10]. A subanalysis of the FIDELIO-DKD trial indicated that, in a similar patient cohort (CKD and T2D), finerenone reduced the risk of new-onset atrial fibrillation and flutter (AFF) [61]. The risk of kidney or cardiovascular events was reduced, irrespective of history of AFF at baseline [61].

## Mineralocorticoid receptor antagonist therapy safety precautions: hyperkalemia

Hyperkalemia has been a major concern with older steroidal MRAs [64]. This is particularly the case in view of the increased use of ACE inhibitors and ARBs in patients with kidney disease and heart failure. The increased risk of hyperkalemia associated with steroidal MRAs also increases with age, which is a concern given the aging population [64]. Meta-analyses have consistently shown a greater risk (approximately two to three times higher) of clinically relevant hyperkalemia in study cohorts receiving steroidal MRA compared with those receiving placebo [65,66]. In a small cohort study of patients aged >75 years, 36% (23/64) of patients developed hyperkalemia (serum potassium ≥5.5 mmol/l) and 10% had severe hyperkalemia (serum potassium >6.0 mmol/l) during the 11-month follow-up period [67].

The large variation in hyperkalemia occurrence seen in studies will have to be addressed by trials with a larger number of participants. The development of nonsteroidal agents may significantly reduce the risk of hyperkalemia [68]. A significant proportion of patients with heart failure who have poor creatinine clearance have been deprived of the benefits of mineralocorticoid receptor blockade, owing to genuine concerns of life-threatening hyperkalemia. Both the RALES [5] and EPHESUS [8] trials excluded patients with serum creatinine >2.5 mg/dl or with initial potassium levels >5.0 mmol/l. Despite these exclusion criteria, the incidence of severe hyperkalemia remained significantly higher in patients receiving steroidal MRA compared with those receiving placebo (1% in the spironolactone arm vs. 2% placebo in RALES) [5].

The limitations of steroidal MRAs prompted extensive basic and clinical research to support the development of nonsteroidal MRAs. In preclinical research, nonsteroidal MRA finerenone has been shown to exhibit weaker potassium-sparing effects than earlier MRAs. Though potassium measurements could not be taken in these animal model studies, results indicated that finerenone was associated with a low risk of hyperkalemia related to renal injury in animal models [69]. Barrera-Chimal *et al*. [69] noted that finerenone was also able to prevent renal dysfunction and tubular injury induced by ischemia–reperfusion in mice.

Finerenone reduced albuminuria in T2D patients with CKD and has been linked to a lower risk of hyperkalemia compared with currently available steroidal MRAs [70] from smaller phase 2 trials with limited numbers. In the FIDELIO-DKD trial, finerenone was shown to be associated with a low incidence of hyperkalemia-related treatment discontinuation (2.3% with finerenone vs. 0.8% with placebo in patients with CVD, and 2.2% with finerenone vs. 1.0% with placebo in patients without CVD) [9]. The FIGARO-DKD trial [10] revealed a lower incidence of hyperkalemia associated with finerenone treatment compared with that in FIDELIO-DKD (10.8% vs. 18.3%) [9]. Given that the risk of hyperkalemia doubles per 15 ml/min decrease in eGFR in patients with CKD, this difference could be explained by the baseline mean eGFR being higher in FIGARO-DKD compared with FIDELIO-DKD (68 vs. 44 ml/min/1.73 m^2^) [9,10]. It is important to note that the encouraging results achieved with finerenone in FIDELIO-DKD were observed despite the fact the lack of restrictions on dietary potassium or potassium supplements, even in patients with hyperkalemia [9]. Overall, newly developed nonsteroidal MRAs indicate promising results and provide novel opportunities for this class of agents.

### Conclusion

Overactivation of mineralocorticoid receptor may stimulate inflammation and fibrosis, resulting in CVD progression. The evidence discussed suggests that MRAs can play an important role in reducing the molecular impact of inflammation and fibrosis, and thereby reduce cardiovascular and renal morbidity. Preclinical findings and randomized clinical trials reinforce this hypothesis by demonstrating that MRAs can reduce the risk of progression of cardiorenal diseases and their associated morbidities. Additionally, recent trials involving patients with T2D and CKD suggest that there may be further, hitherto undiscovered cardiovascular applications for this class of agents. These initial positive cardiovascular effects could potentially be linked to MRA-induced molecular changes and fibrosis reduction. While these initial results may support a new role for nonsteroidal MRAs such as finerenone, additional research is required to validate this hypothesis.

## Acknowledgements

Medical writing support, under the guidance of the authors, was provided by Ananya Das, PhD of integrated medhealth communication (imc), UK, and was funded by Bayer, in accordance with Good Publication Practice (GPP3) guidelines (*Ann Intern Med*. 2022;175(9):1298–1304).

### Conflicts of interest

R.J.C. has consulted and received speaker fees, advisory board participation fees, or investigational grants for Abbott, AstraZeneca Pharmaceuticals, Boehringer Ingelheim, Merck Sharp & Dohme, Pfizer, and Sanofi. All unrelated to this work. J.S.-C. has consulted and received speaker fees, advisory board participation fees, or investigational grants for Abbott, AstraZeneca Pharmaceuticals, Bial, Boehringer Ingelheim, Menarini, Merck Serono, Merck Sharp & Dohme, Novartis, Orion, Pfizer, Sanofi, Servier and Vifor. All unrelated to this work. Medical writing support and article processing charges were funded by Bayer.

## References

[1] Segura AM Frazier OH Buja LM. Fibrosis and heart failure. Heart Fail Rev 2014; 19:173–185. 23124941 10.1007/s10741-012-9365-4

[2] Petrie JR Guzik TJ Touyz RM. Diabetes, hypertension, and cardiovascular disease: clinical insights and vascular mechanisms. Can J Cardiol 2018; 34:575–584. 29459239 10.1016/j.cjca.2017.12.005 PMC5953551

[3] Brown NJ. Contribution of aldosterone to cardiovascular and renal inflammation and fibrosis. Nat Rev Nephrol 2013; 9:459–469. 23774812 10.1038/nrneph.2013.110 PMC3922409

[4] Bomback AS Kshirsagar AV Klemmer PJ. Renal aspirin: will all patients with chronic kidney disease one day take spironolactone? Nat Clin Pract Nephrol 2009; 5:74–75. 19029998 10.1038/ncpneph1004 PMC2845907

[5] Pitt B Zannad F Remme WJ Cody R Castaigne A Perez A. The effect of spironolactone on morbidity and mortality in patients with severe heart failure. Randomized Aldactone Evaluation Study Investigators. N Engl J Med 1999; 341:709–717. 10471456 10.1056/NEJM199909023411001

[6] Kolkhof P Barfacker L. 30 years of the mineralocorticoid receptor: mineralocorticoid receptor antagonists: 60 years of research and development. J Endocrinol 2017; 234:T125–T140. 28634268 10.1530/JOE-16-0600 PMC5488394

[7] Pitt B Remme W Zannad F Neaton J Martinez F Roniker B. Eplerenone, a selective aldosterone blocker, in patients with left ventricular dysfunction after myocardial infarction. N Engl J Med 2003; 348:1309–1321. 12668699 10.1056/NEJMoa030207

[8] Zannad F McMurray JJ Krum H van Veldhuisen DJ Swedberg K Shi H. Eplerenone in patients with systolic heart failure and mild symptoms. N Engl J Med 2011; 364:11–21. 21073363 10.1056/NEJMoa1009492

[9] Bakris GL Agarwal R Anker SD Pitt B Ruilope LM Rossing P. Effect of finerenone on chronic kidney disease outcomes in type 2 diabetes. N Engl J Med 2020; 383:2219–2229. 33264825 10.1056/NEJMoa2025845

[10] Pitt B Filippatos G Agarwal R Anker SD Bakris GL Rossing P. Cardiovascular events with finerenone in kidney disease and type 2 diabetes. N Engl J Med 2021; 385:2252–2263. 34449181 10.1056/NEJMoa2110956

[11] Bakris GL Agarwal R Chan JC Cooper ME Gansevoort RT Haller H. Effect of finerenone on albuminuria in patients with diabetic nephropathy: a randomized clinical trial. JAMA 2015; 314:884–894. 26325557 10.1001/jama.2015.10081

[12] Doggrell SA. Finerenone – are we there yet with a non-steroidal mineralocorticoid receptor antagonist for the treatment of diabetic chronic kidney disease? Expert Opin Pharmacother 2021; 22:1253–1256. 33764251 10.1080/14656566.2021.1904892

[13] Kolkhof P Delbeck M Kretschmer A Steinke W Hartmann E Barfacker L. Finerenone, a novel selective nonsteroidal mineralocorticoid receptor antagonist protects from rat cardiorenal injury. J Cardiovasc Pharmacol 2014; 64:69–78. 24621652 10.1097/FJC.0000000000000091

[14] Kolkhof P Jaisser F Kim SY Filippatos G Nowack C Pitt B. Steroidal and novel non-steroidal mineralocorticoid receptor antagonists in heart failure and cardiorenal diseases: comparison at bench and bedside. Handb Exp Pharmacol 2017; 243:271–305. 27830348 10.1007/164_2016_76

[15] Ito S Kashihara N Shikata K Nangaku M Wada T Okuda Y. Esaxerenone (CS-3150) in patients with type 2 diabetes and microalbuminuria (ESAX-DN): phase 3 randomized controlled clinical trial. Clin J Am Soc Nephrol 2020; 15:1715–1727. 33239409 10.2215/CJN.06870520 PMC7769030

[16] Ito S Shikata K Nangaku M Okuda Y Sawanobori T. Efficacy and safety of esaxerenone (CS-3150) for the treatment of type 2 diabetes with microalbuminuria: a randomized, double-blind, placebo-controlled, phase II trial. Clin J Am Soc Nephrol 2019; 14:1161–1172. 31248950 10.2215/CJN.14751218 PMC6682830

[17] U.S. Food and Drug Administration. KERENDIA (finerenone) tablets, for oral use. Manufactured for Bayer HealthCare Pharmaceuticals Inc., All rights reserved. Manufactured in Germany. Initial U.S. Approval: 2021 Highlights of Prescribing Information. https://www.accessdata.fda.gov/drugsatfda_docs/label/2021/215341s000lbl.pdf. [Accessed June 2023]

[18] European Medicines Agency. Kerendia: EPAR – product information. Manufactured for Bayer HealthCare Pharmaceuticals Inc., All rights reserved. Manufactured in Germany. First published 11 March 2022. https://www.ema.europa.eu/en/documents/product-information/kerendia-epar-product-information_en.pdf. [Accessed June 2023]

[19] Vodosek Hojs N Bevc S Ekart R Piko N Petreski T Hojs R. Mineralocorticoid receptor antagonists in diabetic kidney disease. Pharmaceuticals (Basel) 2021; 14:561. 34208285 10.3390/ph14060561 PMC8230766

[20] Sato A Hayashi K Naruse M Saruta T. Effectiveness of aldosterone blockade in patients with diabetic nephropathy. Hypertension 2003; 41:64–68. 12511531 10.1161/01.hyp.0000044937.95080.e9

[21] Schjoedt KJ Andersen S Rossing P Tarnow L Parving HH. Aldosterone escape during blockade of the renin-angiotensin-aldosterone system in diabetic nephropathy is associated with enhanced decline in glomerular filtration rate. Diabetologia 2004; 47:1936–1939. 15551047 10.1007/s00125-004-1542-0

[22] Bauersachs J Lother A. Mineralocorticoid receptor activation and antagonism in cardiovascular disease: cellular and molecular mechanisms. Kidney Int Suppl (2011) 2022; 12:19–26. 35529088 10.1016/j.kisu.2021.11.001 PMC9073241

[23] Jia G Jia Y Sowers JR. Role of mineralocorticoid receptor activation in cardiac diastolic dysfunction. Biochim Biophys Acta Mol Basis Dis 2017; 1863:2012–2018. 27989961 10.1016/j.bbadis.2016.10.025 PMC5410190

[24] Jia G Habibi J Aroor AR Martinez-Lemus LA DeMarco VG Ramirez-Perez FI. Endothelial mineralocorticoid receptor mediates diet-induced aortic stiffness in females. Circ Res 2016; 118:935–943. 26879229 10.1161/CIRCRESAHA.115.308269 PMC4798906

[25] Jia G Habibi J DeMarco VG Martinez-Lemus LA Ma L Whaley-Connell AT. Endothelial mineralocorticoid receptor deletion prevents diet-induced cardiac diastolic dysfunction in females. Hypertension 2015; 66:1159–1167. 26441470 10.1161/HYPERTENSIONAHA.115.06015 PMC4644106

[26] Gorini S Kim SK Infante M Mammi C La Vignera S Fabbri A. Role of aldosterone and mineralocorticoid receptor in cardiovascular aging. Front Endocrinol (Lausanne) 2019; 10:584. 31507534 10.3389/fendo.2019.00584 PMC6716354

[27] Suthahar N Meijers WC Sillje HHW de Boer RA. From inflammation to fibrosis-molecular and cellular mechanisms of myocardial tissue remodelling and perspectives on differential treatment opportunities. Curr Heart Fail Rep 2017; 14:235–250. 28707261 10.1007/s11897-017-0343-y PMC5527069

[28] Wynn TA Ramalingam TR. Mechanisms of fibrosis: therapeutic translation for fibrotic disease. Nat Med 2012; 18:1028–1040. 22772564 10.1038/nm.2807 PMC3405917

[29] Belden Z Deiuliis JA Dobre M Rajagopalan S. The role of the mineralocorticoid receptor in inflammation: focus on kidney and vasculature. Am J Nephrol 2017; 46:298–314. 29017166 10.1159/000480652 PMC6863172

[30] Epstein M. Aldosterone and mineralocorticoid receptor signaling as determinants of cardiovascular and renal injury: from Hans Selye to the present. Am J Nephrol 2021; 52:209–216. 33857953 10.1159/000515622

[31] Chute M Aujla P Jana S Kassiri Z. The non-fibrillar side of fibrosis: contribution of the basement membrane, proteoglycans, and glycoproteins to myocardial fibrosis. J Cardiovasc Dev Dis 2019; 6:35. 31547598 10.3390/jcdd6040035 PMC6956278

[32] Ferreira JP Verdonschot J Wang P Pizard A Collier T Ahmed FZ. Proteomic and mechanistic analysis of spironolactone in patients at risk for HF. JACC Heart Fail 2021; 9:268–277. 33549556 10.1016/j.jchf.2020.11.010

[33] Rossi MA. Pathologic fibrosis and connective tissue matrix in left ventricular hypertrophy due to chronic arterial hypertension in humans. J Hypertens 1998; 16:1031–1041. 9794745 10.1097/00004872-199816070-00018

[34] Frangogiannis NG. Cardiac fibrosis: cell biological mechanisms, molecular pathways and therapeutic opportunities. Mol Aspects Med 2019; 65:70–99. 30056242 10.1016/j.mam.2018.07.001

[35] Berk BC Fujiwara K Lehoux S. ECM remodeling in hypertensive heart disease. J Clin Invest 2007; 117:568–575. 17332884 10.1172/JCI31044 PMC1804378

[36] Intengan HD Schiffrin EL. Vascular remodeling in hypertension: roles of apoptosis, inflammation, and fibrosis. Hypertension 2001; 38(3 Pt 2):581–587. 11566935 10.1161/hy09t1.096249

[37] Prabhu SD Frangogiannis NG. The biological basis for cardiac repair after myocardial infarction: from inflammation to fibrosis. Circ Res 2016; 119:91–112. 27340270 10.1161/CIRCRESAHA.116.303577 PMC4922528

[38] Simpson SA Tait JF Wettstein A Neher R Von Euw J Schindler O. [Constitution of aldosterone, a new mineralocorticoid]. Experientia 1954; 10:132–133. 13161890 10.1007/BF02158515

[39] Luetscher JA Jr Johnson BB. Observations on the sodium-retaining corticoid (aldosterone) in the urine of children and adults in relation to sodium balance and edema. J Clin Invest 1954; 33:1441–1446. 13211798 10.1172/JCI103022 PMC1072569

[40] Kagawa CM Cella JA Van Arman CG. Action of new steroids in blocking effects of aldosterone and desoxycorticosterone on salt. Science 1957; 126:1015–1016. 13486053 10.1126/science.126.3281.1015

[41] Farrelly RO Howie RN North JD. Use of spironolactone and hydrochlorothiazide in treatment of oedema. Br Med J 1960; 2:339–343. 13821638 10.1136/bmj.2.5195.339 PMC2097506

[42] Garthwaite SM McMahon EG. The evolution of aldosterone antagonists. Mol Cell Endocrinol 2004; 217:27–31. 15134797 10.1016/j.mce.2003.10.005

[43] Corvol P Michaud A Menard J Freifeld M Mahoudeau J. Antiandrogenic effect of spirolactones: mechanism of action. Endocrinology 1975; 97:52–58. 166833 10.1210/endo-97-1-52

[44] Smith WG. Spironolactone and gynaecomastia. Lancet 1962; 280:886.

[45] Greenblatt DJ Koch-Weser J. Gynecomastia and impotence: complications of spironolactone therapy. JAMA 1973; 223:82. 4739107

[46] Menard J. The 45-year story of the development of an anti-aldosterone more specific than spironolactone. Mol Cell Endocrinol 2004; 217:45–52. 15134800 10.1016/j.mce.2003.10.008

[47] Nickisch K Bittler D Casals-Stenzel J Laurent H Nickolson R Nishino Y. Aldosterone antagonists. 1. Synthesis and activities of 6 beta,7 beta:15 beta,16 beta-dimethylene steroidal spirolactones. J Med Chem 1985; 28:546–550. 3989815 10.1021/jm50001a002

[48] Opoku J Kalimi M Agarwal M Qureshi D. Effect of a new mineralocorticoid antagonist mespirenone on aldosterone-induced hypertension. Am J Physiol 1991; 260(2 Pt 1):E269–E271. 1996630 10.1152/ajpendo.1991.260.2.E269

[49] Susic D Varagic J Ahn J Matavelli L Frohlich ED. Long-term mineralocorticoid receptor blockade reduces fibrosis and improves cardiac performance and coronary hemodynamics in elderly SHR. Am J Physiol Heart Circ Physiol 2007; 292:H175–H179. 16905598 10.1152/ajpheart.00660.2006

[50] Arriza JL Weinberger C Cerelli G Glaser TM Handelin BL Housman DE. Cloning of human mineralocorticoid receptor complementary DNA: structural and functional kinship with the glucocorticoid receptor. Science 1987; 237:268–275. 3037703 10.1126/science.3037703

[51] Dietz JD Du S Bolten CW Payne MA Xia C Blinn JR. A number of marketed dihydropyridine calcium channel blockers have mineralocorticoid receptor antagonist activity. Hypertension 2008; 51:742–748. 18250364 10.1161/HYPERTENSIONAHA.107.103580

[52] Grune J Beyhoff N Smeir E Chudek R Blumrich A Ban Z. Selective mineralocorticoid receptor cofactor modulation as molecular basis for finerenone’s antifibrotic activity. Hypertension 2018; 71:599–608. 29437893 10.1161/HYPERTENSIONAHA.117.10360

[53] Pieronne-Deperrois M Gueret A Djerada Z Crochemore C Harouki N Henry JP. Mineralocorticoid receptor blockade with finerenone improves heart function and exercise capacity in ovariectomized mice. ESC Heart Fail 2021; 8:1933–1943. 33742556 10.1002/ehf2.13219 PMC8120350

[54] Jaisser F Tan X Chi S Liu J Wang P Bush M. The non-steroidal mineralocorticoid receptor antagonist KBP-5074 limits albuminuria and has improved therapeutic index compared with eplerenone in a rat model with mineralocorticoid-induced renal injury. Front Pharmacol 2021; 12:604928. 34248613 10.3389/fphar.2021.604928 PMC8264204

[55] Selye H. Protection by a steroid-spirolactone against certain types of cardiac necroses. Proc Soc Exp Biol Med 1960; 104:212–213. 14444794 10.3181/00379727-104-25782

[56] Brilla CG Matsubara LS Weber KT. Antifibrotic effects of spironolactone in preventing myocardial fibrosis in systemic arterial hypertension. Am J Cardiol 1993; 71:12A–16A. 10.1016/0002-9149(93)90239-9 8421998

[57] Cleland JGF Ferreira JP Mariottoni B Pellicori P Cuthbert J Verdonschot JAJ. The effect of spironolactone on cardiovascular function and markers of fibrosis in people at increased risk of developing heart failure: the heart ‘OMics’ in AGEing (HOMAGE) randomized clinical trial. Eur Heart J 2021; 42:684–696. 33215209 10.1093/eurheartj/ehaa758 PMC7878013

[58] Yang P Huang T Xu G. The novel mineralocorticoid receptor antagonist finerenone in diabetic kidney disease: progress and challenges. Metabolism 2016; 65:1342–1349. 27506741 10.1016/j.metabol.2016.06.001

[59] Bower NI Vogrin AJ Le Guen L Chen H Stacker SA Achen MG. Vegfd modulates both angiogenesis and lymphangiogenesis during zebrafish embryonic development. Development 2017; 144:507–518. 28087639 10.1242/dev.146969

[60] Duggan S. Esaxerenone: first global approval. Drugs 2019; 79:477–481. 30806972 10.1007/s40265-019-01073-5

[61] Filippatos G Bakris GL Pitt B Agarwal R Rossing P Ruilope LM. Finerenone reduces new-onset of atrial fibrillation in patients with chronic kidney disease and type 2 diabetes. J Am Coll Cardiol 2021; 78:142–152. 34015478 10.1016/j.jacc.2021.04.079

[62] Wada T Inagaki M Yoshinari T Terata R Totsuka N Gotou M. Apararenone in patients with diabetic nephropathy: results of a randomized, double-blind, placebo-controlled phase 2 dose-response study and open-label extension study. Clin Exp Nephrol 2021; 25:120–130. 32974732 10.1007/s10157-020-01963-z PMC7880964

[63] Sridhar VS Liu H Cherney DZI. Finerenone—a new frontier in renin-angiotensin-aldosterone system inhibition in diabetic kidney disease. Am J Kidney Dis 2021; 78:309–311. 33753202 10.1053/j.ajkd.2021.02.324

[64] House AA. Management of heart failure in advancing CKD: core curriculum 2018. Am J Kidney Dis 2018; 72:284–295. 29478868 10.1053/j.ajkd.2017.12.006

[65] Currie G Taylor AH Fujita T Ohtsu H Lindhardt M Rossing P. Effect of mineralocorticoid receptor antagonists on proteinuria and progression of chronic kidney disease: a systematic review and meta-analysis. BMC Nephrol 2016; 17:127. 27609359 10.1186/s12882-016-0337-0 PMC5015203

[66] Vukadinovic D Lavall D Vukadinovic AN Pitt B Wagenpfeil S Bohm M. True rate of mineralocorticoid receptor antagonists-related hyperkalemia in placebo-controlled trials: a meta-analysis. Am Heart J 2017; 188:99–108. 28577687 10.1016/j.ahj.2017.03.011

[67] Svensson M Gustafsson F Galatius S Hildebrandt PR Atar D. How prevalent is hyperkalemia and renal dysfunction during treatment with spironolactone in patients with congestive heart failure? J Card Fail 2004; 10:297–303. 15309695 10.1016/j.cardfail.2003.10.012

[68] Pei H Wang W Zhao D Wang L Su GH Zhao Z. The use of a novel non-steroidal mineralocorticoid receptor antagonist finerenone for the treatment of chronic heart failure: a systematic review and meta-analysis. Medicine (Baltim) 2018; 97:e0254. 10.1097/MD.0000000000010254 PMC5916685 29668577

[69] Barrera-Chimal J Andre-Gregoire G Nguyen Dinh Cat A Lechner SM Cau J Prince S. Benefit of mineralocorticoid receptor antagonism in AKI: role of vascular smooth muscle Rac1. J Am Soc Nephrol 2017; 28:1216–1226. 28087726 10.1681/ASN.2016040477 PMC5373452

[70] Lytvyn Y Godoy LC Scholtes RA van Raalte DH Cherney DZ. Mineralocorticoid antagonism and diabetic kidney disease. Curr Diab Rep 2019; 19:4. 30673886 10.1007/s11892-019-1123-8

